# A Pilot Study of the Photoprotective Effects of Strawberry-Based Cosmetic Formulations on Human Dermal Fibroblasts

**DOI:** 10.3390/ijms160817870

**Published:** 2015-08-04

**Authors:** Massimiliano Gasparrini, Tamara Yuliett Forbes-Hernandez, Sadia Afrin, José Miguel Alvarez-Suarez, Ana Maria Gonzàlez-Paramàs, Celestino Santos-Buelga, Stefano Bompadre, José Luis Quiles, Bruno Mezzetti, Francesca Giampieri

**Affiliations:** 1 Department of Clinical Sciences, Faculty of Medicine, Polytechnic University of Marche, Ancona 60131, Italy; E-Mails: m.gasparrini@univpm.it (M.G.); tamara.forbe@gmail.com (T.Y.F.-H.); dolla.bihs@gmail.com (S.A.); malvarez@unach.edu.ec (J.M.A.-S.); 2 Area de Nutrición y Salud, Universidad Internacional Iberoamericana (UNINI), Campeche 24040, Mexico; 3 Facultad de Ciencias de la Salud, Universidad Nacional de Chimborazo, Riobamba 060150, Ecuador; 4 Grupo de Investigación en Polifenoles (GIP-USAL), Faculty of Pharmacy, Salamanca University, Campus Miguel de Unamuno, Salamanca E37007, Spain; E-Mails: paramas@usal.es (A.M.G.-P.); csb@usal.es (C.S.-B.); 5 Dipartimento di Scienze Biomediche e Sanità Pubblica, Facoltà di Medicina, Università Politecnica delle Marche Via Ranieri 65, Ancona 60131, Italy; E-Mail: s.bompadre@univpm.it; 6 Department of Physiology, Institute of Nutrition and Food Technology “José Mataix”, Biomedical Research Centre, University of Granada, Granada 18071, Spain; E-Mail: jlquiles@ugr.es; 7 Dipartimento di Scienze Agrarie, Alimentari e Ambientali, Università Politecnica delle Marche, Ancona 60131, Italy; E-Mail: b.mezzetti@univpm.it

**Keywords:** strawberry polyphenols, skin damage, UVA-radiation, topical formulation, cell viability, human dermal fibroblasts

## Abstract

Strawberry polyphenols have been extensively studied over the last two decades for their beneficial properties. Recently, their possible use in ameliorating skin conditions has also been proposed; however, their role in preventing UVA-induced damage in cosmetic formulation has not yet been investigated. Skin is constantly exposed to several environmental stressors, such as UVA radiation, that induce oxidative stress, inflammation and cell death via the production of reactive oxygen species (ROS). In the present study, we assessed the potential photoprotective capacity of different strawberry-based formulations, enriched with nanoparticles of Coenzyme Q_10_ and with sun protection factor 10 (SPF10), in human dermal fibroblasts (HuDe) exposed to UVA radiation. We confirmed that strawberries are a very rich source of polyphenols, anthocyanins and vitamins, and possess high total antioxidant capacity. We also showed that strawberry extracts (25 μg/mL–1 mg/mL) exert a noticeable photoprotection in HuDe, increasing cell viability in a dose-dependent way, and that these effects are potentiated by the presence of CoQ_10red_ (100 μg/mL). We have demonstrated for the first time that the topical use of strawberry extract may provide good photoprotection, even if more in-depth studies are strongly encouraged in order to evaluate the cellular and molecular effects of strawberry protection.

## 1. Introduction

The skin is the largest organ of the body that creates a self-repairing barrier, protecting the body from the most common potentially harmful physical, environmental, and biological insults. Sun UV radiation, together with many other factors to which the skin is constantly exposed, like smoke and microorganisms, is mainly responsible for skin photoaging, hyperplasia, erythema, and even cancer. Excessive exposure of skin to UV radiation can indeed lead to inflammation, oxidative stress, direct and ROS-mediated DNA damage, dysregulation of cellular signalling pathways, and immunosuppression, through the generation of reactive oxygen species (ROS) [1]. To prevent such damage, the skin has developed extremely efficient defence mechanisms: major protective systems include the natural pigment melanin, which absorbs and scatters ultraviolet (UV) radiation, and antioxidant enzymes and nonenzymatic molecules including Coenzyme Q_10_ (CoQ_10_), carotenoids, and vitamin C, that are able to counteract the deleterious effects of oxidative stress to which skin is constantly exposed [2]. Unfortunately, skin is inadvertently exposed to approximately 2/3 of the cumulative erythemal UV dose/year [3] and, beyond the putative photoprotection elicited by dietary compounds via endogenous delivery, topical sunscreen products may significantly contribute to lifelong protection of skin health. In recent years, potential protective activities have been suggested for different natural polyphenols, including green tea polyphenols, grape seed proanthocyanidins, strawberry anthocyanins, silymarin, luteolin, and genistein [4,5]. For example, we found that strawberry polyphenols and vitamins are able to exert a strong protective effect against oxidative- and UVA-induced skin damage, decreasing free radicals, lipid peroxidation, DNA damage, and ameliorating mitochondrial functionality, when incorporated in the culture medium [4,6]. However, the biological activities of polyphenols depend mainly on their bioavailability, which seems to be very low *in vivo* and different among individuals, probably due to differences in the colonic microbiota, making phenol metabolism even more complex to understand and explain [7,8]. For these reasons, there is a greater emphasis on the topical use of natural compounds as an alternative to reduce the harmful effects of UV radiation, maintaining skin well-being, and preventing dermal diseases.

In the present study, a specific combination of a strawberry extract, particularly rich in polyphenols and antioxidant vitamins, and enhanced with CoQ_10_, was used for the first time in a sunscreen preparation to inhibit UVA’s harmful effects on human dermal fibroblasts, evaluating the capacity of this combination to decrease UVA-mediated cell death by MTT assay.

## 2. Results and Discussion

### 2.1. Phenolics, Vitamins and Antioxidant Capacity of Strawberry Extract

Analysis of the *Alba* extract showed that this cultivar is a notable source of polyphenols (2.32 mg gallic acid equivalents (GAEq)/g) and flavonoids (0.61 mg catechin equivalents (CEq)/g) (Table 1); five anthocyanin pigments were detected through HPLC-DAD/ESI-MS analysis, with Pelargonidin (Pg-) and Cyanidin (Cy-) glycosides being the most representative anthocyanin strawberry components (Table 1). Pg-3-glucoside was found in the highest concentration, with 39.7 mg/100 g fresh weight (FW), followed by Pg 3-malonylglucoside, with a concentration of about 6.69 mg/100 g FW.

**Table 1:** Vitamin composition, phytochemical content and antioxidant capacity of strawberry extract.

Parameter	Concentration
Vitamin C (mg/g)	0.58 ± 0.03
β-carotene (μg/100 g)	28.1 ± 0.04
Total phenolic (mg GAEq/g)	2.32 ± 0.02
Total flavonoid (mg CEq/g)	0.61 ± 0.02
*Anthocyanins (mg/100 g)*	
Cy-3-glucoside	3.11 ± 0.02
Pg 3-glucoside	39.7 ± 0.13
Pg 3-rutinoside	3.87 ± 0.16
Pg 3-malonylglucoside	6.69 ± 0.04
Pg 3-acetylglucoside	0.38 ± 0.01
*TAC (μmol Trolox Equivalents TE/g)*	
FRAP	13.62 ± 0.15
ORAC	53.03 ± 0.45

Two essential antioxidants, able to prevent singlet oxygen and free radical-mediated damage [9,10], that are present both in strawberries and in the human skin, are vitamin C and β-carotene. We analysed their content in the extract by HPLC-DAD (Table 1) and found a good concentration of these vitamins (about 58.3 mg/100 g FW and 28.1 μg/100 g FW, respectively) that could contribute to the high antioxidant capacity and free radical scavenging capacity of this cultivar.

The total antioxidant capacity (TAC) of fruit extract was quantified by Ferric Reducing Antioxidant Power (FRAP) and Oxygen Radical Absorbance Capacity (ORAC) assays (Table 1). According to these methods, *Alba* extract showed a high TAC value, with 13.62 and 53.03 μmol TE/g of FW for FRAP and ORAC, respectively. We found that TAC values of the *Alba* cultivar were similar to those previously reported in other strawberry varieties, and confirmed that this cultivar possesses a high TAC [4,6,11,12]. The antioxidant power of fruit is strictly correlated to the presence of efficient oxygen radical scavengers, such as vitamin C and phenolic compounds. In the present study, the concentration of TPC was significantly associated with the TAC, measured respectively with the ORAC and FRAP assays (*r* = 0.81, *p* < 0.001, and *r* = 0.86, *p* < 0.001), and also flavonoids significantly correlated with the TAC of the fruits (*r* = 0.93, *p* = 0.001, and *r* = 0.97, *p* = 0.0001 respectively for ORAC and FRAP assays), as previously demonstrated [13]. Regarding vitamin C, a significant correlation with TAC was expected from its high concentration in strawberries, accounting for a large part of the water-soluble antioxidant capacity. In fact, the vitamin C content significantly correlated with ORAC and FRAP data (*r* = 0.86, *p* = 0.006 and *r* = 0.84, *p* = 0.009, respectively).

### 2.2. Photoprotective Effects of the Formulations

Dietary polyphenols, of which fruits and vegetables are particularly rich, are the most promising group of compounds that can be exploited as chemoprotectants, preventing, delaying or completely halting UV skin damage [14]. Recently, topical application of antioxidant compounds, like retinol and polyphenols, has been shown to exert a protective effect against oxidative skin damage, decreasing free radicals, lipid peroxidation and DNA damage [14,15,16]. Besides polyphenols, another interesting agent that can be used in skin care and sun protection products is CoQ_10_. CoQ_10_ is a lipophilic coenzyme with redox properties embedded in the hydrophilic core of the membranes, widely distributed in almost all living organisms; its function is to transport electrons in the respiratory chain along the inner mitochondrial membrane, during the aerobic respiration and oxidative phosphorylation processes [17]. In addition, CoQ_10_ participates in other important cellular functions, such as disulfide bond formation, cell signals, gene expression, and ROS detoxification. For example, in its reduced form it has strong antioxidant potential, protecting lipids, proteins, and DNA against oxidative stress [18]. Within the skin, its concentration in the epidermis is about 10-fold higher than in the dermis [19] and its delivery can be improved using liposomes or nanoparticles [20,21]. For these reasons, we used Sanomit™ Q_10_ for CoQ_10ox_ and Quinomit™ Q_10_ for CoQ_10red_, which are dispersions of nanoparticles (diameter < 50 nm) of CoQ_10_ in water and are useful for preparing stable liquid-liquid dispersions of various CoQ_10_ concentrations. These two products are absorbed much better and faster compared to ordinary Q_10_ preparations, as previously reported [22,23].

In the present work, we evaluated the photoprotective effects of different formulations containing strawberry extract and CoQ_10_ in UVA exposed HuDe using the MTT assay. This method is based on the evaluation of mitochondrial functionality as an indicator of cell viability, because dehydrogenases present within the mitochondria of only viable cells reduce MTT to purple formazan crystals.

**Figure 1:**
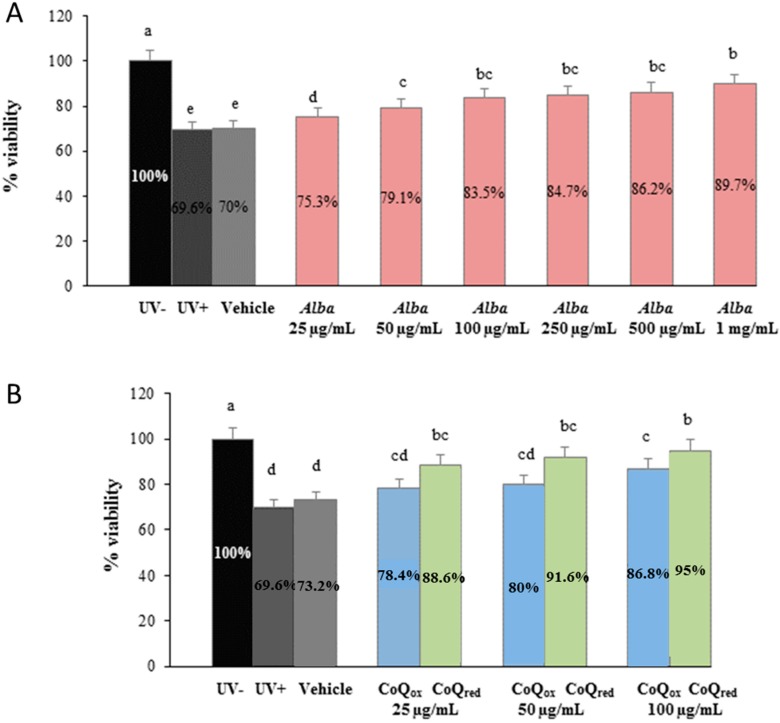
Viability of HuDe exposed or not exposed to UVA (275 kJ/m^2^), assessed using the MTT assay, after 1 h of exposure. HuDe were screened with formulations containing different concentrations of strawberry extracts (**A**), or different concentrations of CoQ_10red_ or CoQ_10ox_ (**B**). Data are expressed as percentage of live cells compared to unexposed controls (UV−). Error bars represent ± S.D; different letters indicate significant difference at *p* ≤ 0.05.

**Figure 2:**
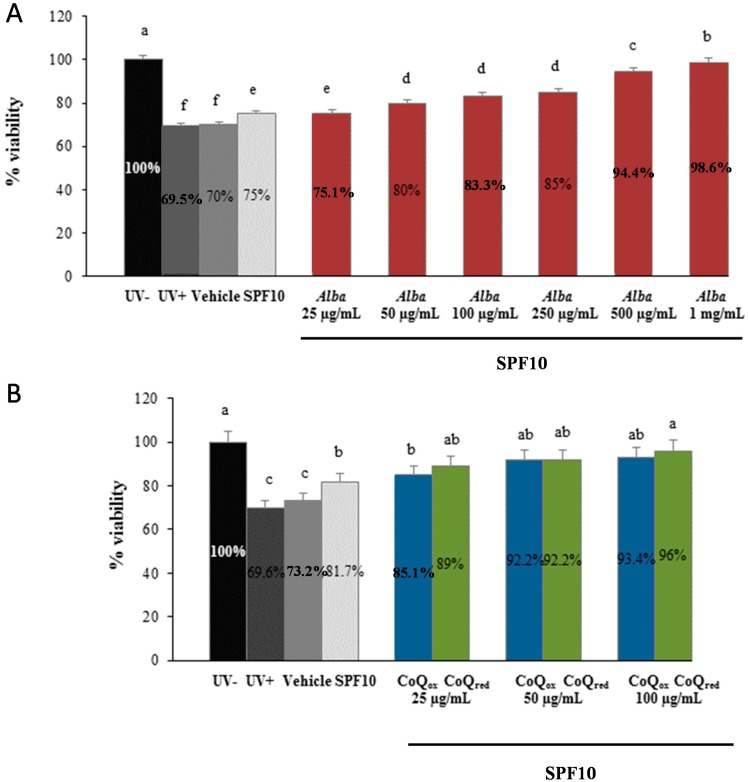
Viability of HuDe exposed or not exposed to UVA (275 kJ/m^2^), assessed using the MTT assay, after 1 h of exposure. HuDe were screened with formulations containing different concentrations of strawberry extracts and SPF10 (**A**), or different concentrations of CoQ_10red_ or CoQ_10ox_ and SPF10 (**B**). Data are expressed as percentage of live cells compared to unexposed controls (UV−). Error bars represent ± S.D; different letters indicate significant difference at *p* ≤ 0.05.

**Figure 3:**
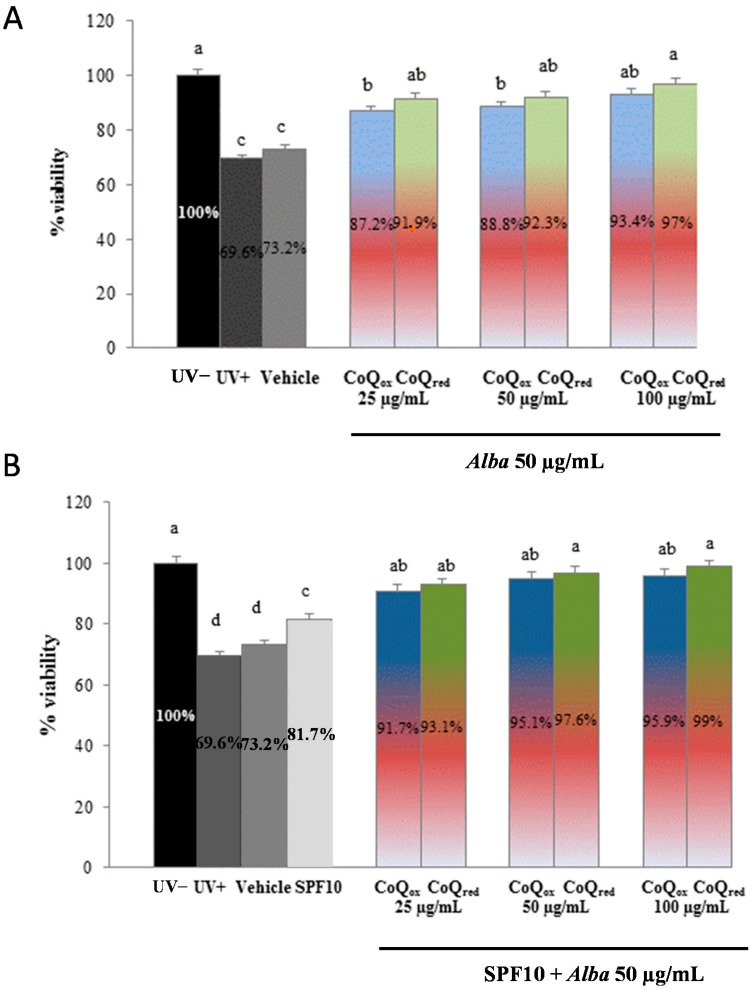
Viability of HuDe exposed or not exposed to UVA (275 kJ/m^2^), assessed using the MTT assay, after 1 h of exposure. HuDe were screened with formulations containing strawberry extract (50 μg/mL) and increasing concentrations of CoQ_10red_ or CoQ_10ox_ without (**A**) and with (**B**) SPF10. Data are expressed as percentage of live cells compared to unexposed controls (UV−). Error bars represent ± S.D; different letters indicate significant difference at *p* ≤ 0.05.

As shown in Figure 1, Figure 2 and Figure 3, UVA exposure produced a significant decrease (35%, *p* ˂ 0.05) in the percentage of live cells in all the samples tested and cells screened by the vehicle alone (polyethylene glycol, PEG) showed a similar behaviour, exerting no significant protection, as expected.

Cells screened by the formulations containing higher concentrations of strawberry extract showed a significantly greater viability (up to 28% for the higher concentration of strawberry) compared to irradiated cells (UV+) (Figure 1A), suggesting a potential photoprotection exerted by strawberry polyphenols. Similar trends were observed for the formulations prepared with CoQ_10red_ or CoQ_10ox_ (Figure 1B).

As expected, a more pronounced photoprotection was found with formulation also containing SPF10: as shown in Figure 2, SPF10 alone exerted only a modest increase in cell viability (7%), with values similar to those of vehicle alone or of irradiated cells (UV+), while there was a significant increase in cell viability with higher concentrations of strawberry extract (Figure 2A) or CoQ_ox_ and CoQ_red_ (Figure 2B), in presence of SPF10.

Finally, we tested the photoprotective capacity of strawberry extract (50 μg/mL) in combination of CoQ_10red_ (25, 50, 100 μg/mL) or CoQ_10ox_ (25, 50, 100 μg/mL) with and without SPF10. As shown in Figure 3, viability was significantly higher in cells screened by the formulation containing strawberry extract and increasing concentrations of CoQ_10_; interestingly, the formulation containing strawberry extract and the higher concentration of CoQ_red_, even in absence of SPF10, was able to prevent the loss in viability restoring it to levels comparable to those of non-irradiated samples (Figure 3A). This photoprotection was more pronounced in the presence of SPF10, as expected (Figure 3B).

## 3. Experimental Section

### 3.1. Standards and Reagents

Three common UV-filters were kindly supplied by BASF (Italy): *bis*-ethylhexyloxyphenol methoxyphenyl triazine, diethylamino hydroxybenzoyl hexyl benzoate and Octocrylene. Liquid CoQ_red_ (Quinomit^®^) and CoQ_ox_ (Sanomit^®^) were kindly provided by MSE Pharmazeutika GmbH (Bad Homburg, Germany).

### 3.2. Strawberry Samples

Strawberry fruits of the “*Alba*” variety were collected in the experimental fields of the Agricultural Faculty of Università Politecnica Marche. Strawberry samples were hand-picked at the same day-time in different days, corresponding to the ripening times of the selected clone, and were selected for homogeneity, avoiding unripe, wounded or shriveled samples. Within 2 h after harvest, whole strawberries were stored at −20 °C before analyses. For evaluation of total antioxidant capacity, total phenolic, anthocyanin and flavonoids contents, a methanolic extract was prepared via homogenization, as previously described [11]. Frozen strawberries were thawed for 60 min at 4 °C. Ten gram aliquots of the samples were added to 100 mL of extraction solution, consisting of methanol/Milli-Q water/concentrated formic acid (80:20:0.1 *v*/*v*/*v*), and strawberries were homogenized using an Ultraturrax T25 homogeniser (Janke & Kunkel, IKA Labortechnik, Staufen, Germany) at medium-high speed for 2 min. Extraction was maximized by stirring the suspension for 2 h in the dark at room temperature, then tubes were centrifuged at 3500 rpm for 15 min in two sequential times, to sediment solids. Supernatant was filtered through a 0.45 μm Minisart filter (PBI International, Milan, Italy), transferred to 5.0 mL amber glass vials and stored at −20 °C until analysis. Immediately before the preparation of formulations, this supernatant was thawed at room temperature, concentrated through a rotary evaporator, and used for the cosmetic preparation.

For vitamin C analysis, the extracting solution consisted in Milli-Q water containing 5% meta-phosphoric acid and 1 mM EDTA. Vitamin C was extracted by sonication of 1 g of frozen strawberries in 4 mL of extracting solution for 5 min, after a previous homogenization using an Ultraturrax T25 homogenizer (Janke & Kunkel, IKA Labortechnik) at medium-high speed for 2 min. After the ultra-sound assisted extraction, the solution was centrifuged at 2500 rpm for 10 min at 4 °C, the supernatant was filtered through a 0.45 μm PTFE filter and immediately analyzed [24]. For β-carotene assessment, 5 g of strawberry were added to 30 mL of acetone, sonicated for 15 min, stirred for 1 h in the dark at room temperature and centrifuged at 9000 rpm at 4 °C for 15 min twice [6]. For saponification, 50 mL of methanolic KOH (10%) was added, left overnight at room temperature and the extract was transferred to 100 mL of petroleum ether and the organic layer dried. The dried residue was dissolved in hexane and filtered through a 0.45 μm membrane filter.

For anthocyanin evaluation in HPLC-DAD-MS, the extract was passed through a C-18 SepPaks Vac 6cc cartridge (Waters, Milan, Italy), the column was washed with 15 mL of ultrapure water to remove sugars and more polar substances while anthocyanin pigments were eluted with 5 mL of methanol: 0.1% trifluoracetic acid (95:5) [4]. The anthocyanin fraction was concentrated under vacuum in a rotary evaporator and samples were diluted with ultrapure water and filtered through a 0.45-mm membrane filter (PBI international, Milan, Italy) prior to the HPLC analysis.

### 3.3. Total Phenolic Content (TPC)

The TPC of strawberry extracts was determined using the Folin-Ciocalteu colorimetric method, as modified by Slinkard and Singleton [25]. Briefly, 100 μL of sample (Milli-Q water, water diluted strawberry extracts or gallic acid standard solutions) were added to 500 μL of Folin-Ciocalteau reagent previously water diluted (1/10) and kept at 4 °C, in the dark. The mixture was incubated for 1–8 min at room temperature, then 400 μL of 0.7 M sodium carbonate (CNa_2_O_3_) was added and the mixture vortexed. The solution was incubated for 2 h at room temperature (~23 °C), in the dark, then the specific absorbance was read at 760 nm, after zeroing the spectrophotometer with blank. Calibration was obtained by plotting the known gallic acid (GA) standard solution concentrations *versus* the corresponding absorbance at 760, and final results were expressed as milligrams of gallic acid equivalents per gram of fresh weight of strawberry (mg GAEq/g). Data were reported as a mean value of three replications.

### 3.4. Total Flavonoids Content

Total flavonoid content was determined by using a colorimetric method described previously [26]. Briefly, 250 μL of reagent (water, strawberry extract or (+)-Catechin standard solution) were mixed to 1.25 mL of Milli-Q water in a test tube, following by addition of 75 μL of a 5% sodium nitrate (NaNO_2_) solution. After 6 min, 150 μL of a 10% aluminum chloride hexahydrate (AlCl_3_·6H_2_O) solution was added to the mixture, and allowed to stand for 5 min. Then, 500 μL 1 M sodium hydroxide (NaOH) were added, the mixture was brought to 2.5 mL with Milli-Q water and mixed well, and the absorbance was immediately read at 510 nm against blank. Results were expressed as mg of catechin equivalents per gram of fresh weight of strawberry (mg CEq/g), in three replicates.

### 3.5. Vitamin C Content

Vitamin C was measured through HPLC analysis immediately after the extraction procedure. The HPLC system comprised a Jasco PU-2089 Plus controller and a Jasco UV-2070 Plus ultraviolet (UV) detector set at absorbance of 260 nm. The HPLC column used was a Supelcosil LC8 150 × 4.6 mm. The elution was isocratic with 50 mM potassium phosphate (KH_2_PO_4_) at pH 3.2 at a rate of 0.8 mL·min^−1^ [27]. Quantification of vitamin C was carried out through a comparison with pure vitamin C calibration curve. Results were expressed as mg vitamin C per g FW (mg/g), in three replicates.

### 3.6. β-Carotene Content

The β-carotene content was determined using the HPLC-DAD method previously reported [28]. Samples were injected in the HPLC-DAD system (Shimadzu Corp., Kyoto, Japan) furnished with a Waters 600 controller and a Waters 996 photodiode array (PDA) detector. A Supelcosil™ LC-18 (150 × 4.6 mm) was used as analytical column and a solution of acetonitrile-methanol-ethyl acetate (88:10:2 *v*/*v*/*v*) in isocratic gradient flow at a rate of 1.0 mL·min^−1^ was employed as the mobile phase. A β-carotene standard was used for quantification and results were expressed as μg β-carotene per 100 g of FW.

### 3.7. Total Antioxidant Capacity

Two methods were used for the determination of the TAC of strawberry extracts: the ORAC and the FRAP.

#### 3.7.1. ORAC

The ORAC assay is based on the procedure previously described [29], where free radicals are produced by the radical generator AAPH, which oxidize the fluorescent compound fluorescein leading to loss in fluorescence. All reagents were prepared in phosphate buffer (pH 7.0, 75 mM) and Trolox (25–150 μmol) was used as standard. The hydroalcoholic extracts were suitably diluted in the phosphate buffer. Each well of a 96 well microplate contained, in a final volume of 200 μL assay solution, 150 μL of fluorescein (0.08 μM) and 25 μL of the undiluted strawberry extract, preincubated for 10 min at 37 °C, then 25 μL of AAPH (150 mM) were added. After addition of AAPH, the plate was shaken automatically for 3 s and the fluorescence was measured every 2 min for 120 min with emission and excitation wavelengths of 530 and 485 nm, respectively, using a microplate fluorescence reader (Synergy™ Multi-Detection Microplate Reader; Bio-Tek^®^, Instruments, Inc., Winooski, VT, USA) that was maintained at 37 °C. The ORAC values were calculated as area under the curve (AUC) and expressed as micromole of Trolox equivalent per gram of FW of strawberry (μmol TE/g of FW).

#### 3.7.2. FRAP

The FRAP assay was carried out according to the protocol proposed by Deighton *et al*. 2000 [13], with slight modifications [30]. The antioxidant capacity of the sample solution was determined by its ability to reduce the pale rust ferric solution to the blue ferrous solution, using Trolox as a reference standard. The FRAP reagent solution was prepared daily immediately prior to procedure, by combining ten volumes of sodium acetate (300 mM, pH 3.6) with one volume of TPTZ (10 mM in HCl 40 mM) and one volume of ferric chloride (20 mM) aqueous solutions. The FRAP reagent was stable for at least 2 h at room temperature. Briefly, 100 μL of blank Trolox standard or 10-fold Milli-Q water diluted strawberry extract were added to 900 μL FRAP reagent into 1.5 mL eppendorfs. The mixture was then quickly vortexed for 15 s and allowed to rest for 4 min. Absorbance of the solution was read at 593 nm (Beckman spectrophotometer, DU644 model) against the blank. Each sample was analyzed in three replicates and FRAP results were expressed as μmol Trolox equivalents/g FW (μmol TE/g).

### 3.8. HPLC-MS

HPLC-DAD/ESI-MS^n^ analysis was performed using a Hewlett-Packard 1200 series liquid chromatography (Agilent Technologies, Waldbronn, Germany) coupled to an HP ChemStation data-processing station, as previously described [3]. A 150 mm × 4.6 mm i.d., 5 μm Aqua C18 column (Phenomenex, Torrance, CA, USA) thermostated at 35 °C was used as stationary phase and the mobile phase was: (A) 0.1% trifluoroacetic acid in water; and (B) HPLC-grade acetonitrile, using the following gradient: isocratic 10% B for 5 min, 10%–15% B over 15 min, isocratic 15% B for 5 min, 15%–18% B over 5 min, and 18%–35% B over 20 min, with a flow rate of 0.5 mL·min^−1^. Double online detection was performed by the combined use of a diode array spectrophotometer (DAD) and an API 3200 Qtrap MS equipped with an ESI source and a triple quadrupole-ion trap mass analyzer (Applied Biosystems, Darmstadt, Germany) controlled by Analyst 5.1 software (Merck, Cairo, Egypt) and connected to the HPLC system via the DAD cell outlet. Zero grade air served as the nebulizer gas (50 psi) and turbo gas for solvent drying (600 °C, 40 psi). Nitrogen served as the curtain (10 psi) and collision gas (medium). Quadrupoles were set at unit resolution. The ion spray voltage was set at 5000 V in the positive mode. Enhanced Ms (EMS) was employed to detect all the ions. Settings used were: declustering potential (DP) 41 V, entrance potential (EP) 7.5 V and collision energy (CE) 10 V. Enhanced product ion (EPI) mode was further performed in order to obtain the fragmentation pattern of the parent ion(s) in the previous experiment using the following parameters: DP 41 V, EP 7.5 V, CE 25 V, and collision energy spread (CES) 0 V. Mass spectrometry data were acquired in the positive mode and ACYs were quantified using the external standards of Cy-3-glucoside and of Pg-3-glucoside (at 520 nm). Results were expressed as mg ACY per 100 g FW (mg/100 g); every strawberry sample was assayed in triplicate.

### 3.9. Cell Culture

Fibroblasts (primary cell cultures of HuDe) were obtained from the American Type Culture Collection (Manassas, VA, USA), cultured in DMEM (Carlo Erba Reagents, Milan, Italy) supplemented with 10% foetal bovine serum (FBS) and antibiotics (100 IU/mL penicillin and 100 μg/mL streptomycin), at 37 °C in a humidified atmosphere with 5% CO_2_.

### 3.10. Filter and Formulation Preparation

An UV filter combination often used in SPF10 sunscreen products was chosen for this study and prepared as follows: 2% *bis*-ethylhexyloxyphenol methoxyphenyl triazine (*w*/*w*), 2% diethylamino hydroxybenzoyl hexyl benzoate (*w*/*w*) and 2% Octocrylene (*w*/*w*) were mixed in PEG and vortexed for 30 min at room temperature in the dark. Different formulations, containing only PEG or PEG + SPF10, were then enriched with different concentrations of strawberry extract (25 μg/mL–1 mg/mL), or reduced CoQ_10_ (25–100 μg/mL) or oxidized CoQ_10_ (25–100 μg/mL). Different combinations of strawberry + CoQ_10red_ and strawberry + CoQ_10ox_ were also prepared.

### 3.11. UV Treatment

The UVA irradiating source was a Saalmann Palma Plant Box Universal (Biosonic, Bologna, Italy) equipped with a 320 W ozone-free lamp, UV type 3. The source delivered 15 mW/cm^2^ between 315 and 400 nm at a distance of 20 cm from the cell cultures. It was always pre-run for 10 min to allow the output to stabilize. The incident dose of UVA received by the samples was 275 kJ/m^2^, *i.e.*, the dose approximately equivalent to about 90 min of sunshine at the French Riviera (Nice) in summer at noon [31].

Cells grown on a 96-well plate for UVA irradiation were washed twice with phosphate buffered saline (PBS) and covered with a thin layer of PBS prior to exposure. Each formulation (2 mg/cm^2^) was spread onto quartz-bottom petri dishes (Hubei Yunsheng Quarts Products Co., Ltd., China) of exactly the same dimensions as the cell culture plates and the dishes were placed on top of the wells prior to irradiation. Cells were placed on a brass block embedded in ice in order to reduce the temperature and, hence, the evaporation during exposure, which could eventually dry out the medium. The cells were then either not exposed (negative control), or exposed to the UVA source (positive control), as previously described, for 60 min, because longer exposure times showed a significant loss of vitality (data not shown).

### 3.12. Cell Viability Assay

After exposure to UVA as described above, PBS was removed and replaced with 200 μL of fresh culture medium and cells were immediately analyzed for viability using the MTT assay [32]. After incubation, fibroblasts were incubated with a salt solution of MTT at a concentration of 0.5 mg/mL for 2 h at 37 °C. The medium was then removed and the crystals were dissolved in DMSO. The optical density of the suspension was read at 550 nm using a microplate reader (Synergy HT, Biotek, Winooski, VT, USA). Cell viability was expressed as a percentage of live cells compared to the unexposed control. The data reported represent average values from at least three independent experiments.

### 3.13. Statistical Analysis

All results are expressed as means ± SD. Statistical analysis was performed using the one-way ANOVA and Tukey’s *post hoc* Test correlations were calculated according to Pearson’s Test. *p* ≤ 0.05 was considered as significant.

## 4. Conclusions

Our results confirm that strawberries possess a high antioxidant capacity, as well as an important anthocyanin and vitamin content, which results in a protective effect on skin cells against UVA induced damage. With this preliminary study we show, for the first time, the possibility of using strawberry extract in sun protection products, even if more studies are strongly encouraged in order to highlight the cellular and molecular mechanisms through which strawberry bioactive compounds exert photoprotection. The next step will be to define the cellular uptake, enzymatic biotransformation, and localization of strawberry compounds in different subcellular districts, to confirm the potential use of strawberry polyphenols in topical/cosmetic treatment as a natural source of antioxidants against skin damage.
